# Clinical practice guidelines for esophagogastric junction cancer: Upper GI Oncology Summit 2023

**DOI:** 10.1007/s10120-023-01457-3

**Published:** 2024-02-22

**Authors:** Yuko Kitagawa, Satoru Matsuda, Takuji Gotoda, Ken Kato, Bas Wijnhoven, Florian Lordick, Pradeep Bhandari, Hirofumi Kawakubo, Yasuhiro Kodera, Masanori Terashima, Kei Muro, Hiroya Takeuchi, Paul F. Mansfield, Yukinori Kurokawa, Jimmy So, Stefan Paul Mönig, Kohei Shitara, Sun Young Rha, Yelena Janjigian, Daisuke Takahari, Ian Chau, Prateek Sharma, Jiafu Ji, Giovanni de Manzoni, Magnus Nilsson, Paulo Kassab, Wayne L. Hofstetter, Elizabeth Catherine Smyth, Sylvie Lorenzen, Yuichiro Doki, Simon Law, Do-Youn Oh, Khek Yu Ho, Tomoyuki Koike, Lin Shen, Richard van Hillegersberg, Hisato Kawakami, Rui-Hua Xu, Zev Wainberg, Naohisa Yahagi, Yeong Yeh Lee, Rajvinder Singh, Min-Hee Ryu, Ryu Ishihara, Zili Xiao, Chika Kusano, Heike Irmgard Grabsch, Hiroki Hara, Ken-ichi Mukaisho, Tomoki Makino, Mitsuro Kanda, Eisuke Booka, Sho Suzuki, Waku Hatta, Motohiko Kato, Akira Maekawa, Akihito Kawazoe, Shun Yamamoto, Izuma Nakayama, Yukiya Narita, Han-Kwang Yang, Masahiro Yoshida, Takeshi Sano

**Affiliations:** 1 Department of Surgery, Keio University School of Medicine, 35 Shinanomachi, Shinjuku-Ku, Tokyo, 160-8582 Japan; 2 Department of Gastroenterology, Cancer Institute Hospital of Japanese Foundation for Cancer Research, Tokyo, Japan; 3 Department of Gastrointestinal Medical Oncology, National Cancer Center Hospital, Tokyo, Japan; 4 Department of Head and Neck, Esophageal Medical Oncology, National Cancer Center Hospital, Tokyo, Japan; 5 Department of Surgery, Erasmus University Medical Center Rotterdam, Rotterdam, The Netherlands; 6 Department of Oncology and University Cancer Center Leipzig, Leipzig University Medical Center, Comprehensive Cancer Center Central, Leipzig, Jena, Germany; 7 Department of Gastroenterology, Portsmouth University Hospital NHS Trust, Portsmouth, UK; 8 Department of Gastroenterological Surgery, Nagoya University Graduate School of Medicine, Nagoya, Japan; 9 Division of Gastric Surgery, Shizuoka Cancer Center, Shizuoka, Japan; 10 Department of Clinical Oncology, Aichi Cancer Center Hospital, Nagoya, Japan; 11 Department of Surgery, Hamamatsu University School of Medicine, Hamamatsu, Japan; 12 Surgical Oncology, University of Texas, MD Anderson Cancer Center, Houston, USA; 13 Department of Gastroenterological Surgery, Graduate School of Medicine, Osaka University, Suita, Japan; 14 Department of Surgery, Yong Loo Lin School of Medicine, National University of Singapore, Singapore, Singapore; 15 Upper-GI-Surgery University Hospital of Geneva, Rue Gabrielle-Perret-Gentil 4, Geneva, Switzerland; 16 Department of Gastrointestinal Oncology, National Cancer Center Hospital East, Kashiwa, Japan; 17 Medical Oncology, Yonsei Cancer Center, Yonsei University College of Medicine, Seoul, Republic of Korea; 18 Department of Medicine, Solid Tumor Gastrointestinal Oncology, Memorial Sloan Kettering Cancer Center, New York, USA; 19 Gastroenterological Chemotherapy, Cancer Institute Hospital of Japanese Foundation for Cancer Research, Tokyo, Japan; 20 Department of Medicine, Royal Marsden Hospital, London, UK; 21 Division of Gastroenterology, School of Medicine and VA Medical Center, University of Kansas, Kansas, USA; 22 Department of Gastrointestinal Surgery, Peking University Cancer Hospital, Beijing, China; 23 Department of Surgery, Dentistry, Maternity and Infant, University of Verona, Verona, Italy; 24 Division of Surgery and Oncology, Department of Clinical Science, Intervention and Technology, Department of Upper Abdominal Diseases, Karolinska University Hospital, Stockholm, Sweden; 25 Gastroesophageal Surgery, Santa Casa of Sao Paulo Medical School, São Paulo, Brazil; 26 Department of Thoracic and Cardiovascular Surgery, University of Texas, MD Anderson Cancer Center, Houston, USA; 27 Oxford NIHR Biomedical Research Centre, Churchill Hospital, Oxford, UK; 28 Department of Hematology and Oncology, Klinikum Rechts Der Isar Munich, Munich, Germany; 29 Department of Surgery, School of Clinical Medicine, The University of Hong Kong, Hong Kong, China; 30 Medical Oncology, Department of Internal Medicine, Seoul National University Hospital, Cancer Research Institute, Seoul National University College of Medicine, Integrated Major in Innovative Medical Science, Seoul National University Graduate School, Seoul, Republic of Korea; 31 National University of Singapore, Singapore, Singapore; 32 Division of Gastroenterology, Tohoku University Graduate School of Medicine, Sendai, Japan; 33 Department of Gastrointestinal Oncology, Peking University Cancer Hospital, Beijing, China; 34 Department of Upper Gastrointestinal Surgery, University Medical Center Utrecht, Utrecht, The Netherlands; 35 Department of Medical Oncology, Faculty of Medicine, Kindai University, Higashiosaka, Japan; 36 Department of Medical Oncology, Sun YAT-Sen University Cancer Center, Guangzhou, China; 37 Gastrointestinal Medical Oncology, David Geffen School of Medicine, University of California Los Angeles, Los Angeles, USA; 38 Cancer Center, Keio University School of Medicine, Tokyo, Japan; 39 School of Medical Sciences, Universiti Sains Malaysia, Penang, Malaysia; 40 Department of Gastroenterology, Lyell McEwin Hospital, Elizabeth Vale, Australia; 41 Department of Oncology, Asan Medical Center, University of Ulsan College of Medicine, Ulsan, South Korea; 42 Gastrointestinal Oncology, Osaka International Cancer Institute, Osaka, Japan; 43 Digestive Endoscopic Unit, Huadong Hospital Affiliated to Fudan University, Shanghai, China; 44 Department of Gastroenterology, Kitasato University School of Medicine, Sagamihara, Japan; 45 Department of Pathology, GROW School for Oncology and Reproduction, Maastricht University Medical Center+, Maastricht, The Netherlands; 46 Pathology & Data Analytics, Leeds Institute of Medical Research at St. James’s, University of Leeds, Leeds, UK; 47 Gastroenterology, Saitama Cancer Center, Saitama, Japan; 48 Education Center for Medicine and Nursing, Shiga University of Medical Science, Otsu, Japan; 49 Department of Gastroenterology, International University of Health and Welfare Ichikawa Hospital, Ichikawa, Japan; 50 Center for Diagnostic and Therapeutic Endoscopy, Keio University School of Medicine, Tokyo, Japan; 51 Department of Gastroenterology, Osaka Police Hospital, Osaka, Japan; 52 Department of Surgery, Seoul National University, Seoul, Republic of Korea; 53 Department of Hepato-Biliary-Pancreatic and Gastrointestinal Surgery, School of Medicine, International University of Health and Welfare, Otawara, Japan; 54 Gastroenterological Surgery, Cancer Institute Hospital of Japanese Foundation for Cancer Research, Tokyo, Japan

**Keywords:** Esophagogastric junction cancer, Guidelines, Multidisciplinary treatment

## Introduction

### Background and purpose

While advances in multimodal therapies have helped in improving the outcome of patients with cancer of the esophagogastric junction (EGJ), current treatment strategies have become increasingly diverse. Optimal strategies vary across countries and institutions. However, in the coming years, it is important for oncologists to take a global and comprehensive view on the treatment of EGJ cancer. To meet this objective, the Upper GI Oncology Summit was organized: an international consensus meeting on EGJ cancer for establishing international clinical practice guidelines at the International Gastric Cancer Conference (IGCC) 2023.

The primary objective of these guidelines is to provide clinicians with information that would guide them to make informed choices on the diagnosis and curative treatment of EGJ cancer (excluding non-epithelial malignant tumors and metastatic malignant EGJ tumors). Furthermore, these guidelines are also intended as an aid for healthcare professionals and patients and caregivers to help them understand the fundamental principles of the diagnosis and treatment of EGJ cancer.

### Target users

The main target users of the guidelines are general clinicians and physicians specializing in the diagnosis and treatment of EGJ cancer. The guidelines also provide useful information to healthcare professionals other than physicians involved in the diagnosis and treatment of EGJ cancer and also for patients and their family members.

### Target patients

The guidelines are intended for the management of adult patients with EGJ cancer.

### Disclosures

The guidelines are not intended for standard diagnosis and treatment covered by the national health insurance system in any specific country. Emphasis has been placed on evidence obtained from patients with EGJ cancer and attention was also paid to the background and indications for treatment of EGJ cancer around the world. The guidelines are intended to provide physicians a standard for diagnosis and treatment but not to force them to provide a specific diagnostic test and/or treatment. Since treatment of EGJ cancer is often multidisciplinary, individualized and requires specific expertise and equipment (including endoscopy, surgery, chemotherapy, radiotherapy and intensive care unit), the diagnosis/treatment should be determined in accordance with the patient’s condition and local health care setting. Therefore, the treating physicians and not the committee of the present guideline is responsible for appropriate patient management.

## Method of development of the guidelines

The guidelines were prepared by referring to the Minds Manual for Guideline Development 2017 and 2020 [1], issued by the Information Division of the Medical Information Network Distribution Service EBM (Minds) and the Japan Council for Quality Health Care under the supervision by Masahiro Yoshida, a member of the GRADE working group.

## Preparation of clinical questions (CQs) and search of the literature

The organizing committee was established in 2021. Subsequently, 49 expert panel members (EPM) were selected from the fields of Surgery, Endoscopy, Medical Oncology, and Pathology. After meticulous discussions among the EPMs, 4 CQs from each field were proposed. After further discussion, 2 CQs were merged into one CQ in both the Endoscopy and Medical Oncology fields, and the total of 10 CQs were then reviewed in the voting process (Table 1). The International Medical Information Center (https://www.imic.or.jp/english/services/) was entrusted with a systematic search of the literature published on the treatment of EGJ cancer which included online publication, using keywords extracted from the CQs. The details are described in Supplementary Table 1. The MEDLINE and Cochrane Library databases were used to search for articles in the English language. Moreover, a manual search was also conducted for articles/ papers that had escaped retrieval by the systematic search and for those published after July 2022, as needed, based on information provided by the systematic review (SR) team and EPMs. Consequently, 2 additional articles were included in the analysis by the manual search [2, 3].

- (1) Inclusion criteria

**Table 1:** Clinical questions and recommendations

Surgery CQ1 Is the dissection of mediastinal and suprapancreatic lymph node stations required for EGJ cancers with 2–4 cm esophageal invasion? *Recommendation* It is weakly recommended to dissect the lower mediastinal and suprapancreatic lymph node stations during surgery in patients with EGJ cancers showing an esophageal invasion length of 2–4 cm
Surgery CQ2 Is it recommended to dissect the same lymph node region of EGJ squamous cell carcinoma and EGJ adenocarcinoma? *Recommendation* It is weakly recommended to conduct a similar degree (location and station) of dissection of lymph nodes, regardless of the histological type, during surgery for cT2 or deeper EGJ cancers
Surgery CQ3 Is minimally invasive surgery recommended for EGJ cancer when a transthoracic approach is indicated? *Recommendation* It is weakly recommended to conduct thoracoscopic (robotic) esophagectomy in patients with resectable EGJ cancer (adenocarcinoma, squamous cell carcinoma) compared to open esophagectomy when a transthoracic approach is indicated
Surgery CQ4 Is surgical resection recommended for gastroesophageal junction cancer with oligo-metastasis? *Recommendation* It is weakly recommended to conduct a surgical resection after chemotherapy in carefully selected EGJ cancer patients presenting with oligo-metastases
Endoscopy CQ1 Is WLE alone recommended for the detection of superficial neoplasia (cancer/HGD) at the GEJZ as compared to WLE combined with image-enhanced endoscopy? *Recommendation* It is weakly recommended to use WLE alone for the detection of esophagogastric junction adenocarcinoma, except in high-risk patients such as those with Barrett’s esophagus
Endoscopy CQ2 Is WLE useful to determine the extent of superficial neoplasia (cancer/HGD) at the GEJZ? *Recommendation* It is weakly recommended to use other modalities, such as IEE, in addition to WLE alone, for determining the lateral extent of superficial neoplasia at the GEJZ
Endoscopy CQ3 What are the criteria for curative resection of neoplasia at the GEJZ? *Recommendation* There are data to recommend endoscopic resection with curative intent for superficial neoplasia of GEJZ. It is weakly recommended that endoscopic resection is considered as curative if the following criteria are fulfilled: the tumor is either (i) an intramucosal carcinoma, or (ii) carcinoma with a submucosal invasion depth of < 500 um; and the tumor diameter < 3 cm; negative resection margins; with no evidence of lymphovascular invasion or poorly differentiated components
Medical oncology CQ1 Is it recommended to adopt chemotherapy for gastric adenocarcinoma to esophageal adenocarcinoma and esophagogastric junction cancer? *Recommendation* It is weakly recommended to use the same chemotherapy regimens as those established for patients with unresectable advanced or recurrent gastric adenocarcinoma in patients with esophagogastric junction adenocarcinoma and esophageal adenocarcinoma
Medical oncology CQ2 What is the optimal perioperative treatment for resectable, locally advanced esophagogastric junction cancer? *Recommendation* Based on the both Eastern and Western evidence, it is weakly recommended to provide perioperative chemotherapy or neoadjuvant chemoradiotherapy for patients with resectable advanced esophagogastric junction cancer. However, the upfront surgery followed by adjuvant chemotherapy may also be acceptable for patients with advanced esophagogastric junction cancer, as for patients with advanced gastric cancer
Medical oncology CQ3 What biomarkers are recommended to be tested before first line for unresectable case? *Recommendation* It is strongly recommended to evaluate the expression statuses of HER2, PD-L1 CPS, MSI, and claudin 18.2 prior to first-line chemotherapy in patients with unresectable esophagogastric junction adenocarcinoma
CQ, clinical question; EGJ, esophagogastric junction; WLE, white light endoscopy; HGD, high grade dysplasia; GEJZ, gastroesophageal junction zone; IEE, image enhancement endoscopy; CPS, combined positive score; MSI, microsatellite instability

Randomized controlled trials and observational studies conducted in adult patients with EGJ cancer were adopted, in principle. Only papers written in English were adopted. Contents of other documents, such as expert reviews and guidelines from other countries, were also reviewed in detail as reference data, although none of these was used as primary evidence.

- (2) Exclusion criteria

Genetic studies and experimental studies in laboratory animals were excluded.

## Systematic review procedure

The SR was conducted independent of the EPMs by member of experienced SR team who were selected by organizing committee based on the previous SR experience. For each of the CQs, the outcomes in terms of the balance between the benefits and risks were extracted and the level of importance thereof was presented. Each retrieved article was subjected to primary and secondary screening, summarized, and then assessed for bias, besides classification of the study design. For each outcome in terms of the benefits and risks, individual papers were considered and the results evaluated as a whole body of evidence, and the strength (certainty) of evidence was determined according to the Minds Manual for Guideline Development 2017 and 2020 [1].

## Determination of the strength of the recommendations

The EPMs drafted the recommendation statements based on the results of the systematic review. The strength of each recommendation was examined on the ground of the certainty of evidence, patient preferences, benefits and risks, and cost evaluation. As for the method of arriving at a consensus, the first vote was held via email in January 2023. During the voting, the EPMs were not allowed to share their results with each other.

The strength of each recommendation was expressed in two directions × 2 steps as follows:

1. Conduct or non-conduct is “strongly recommended.”
2. Conduct or non-conduct is “weakly recommended.”

Based on the results of the first vote, the SR team and EPMs revised the recommendation statements for cases with an agreement rate of lower than 70%. Then the second vote was held on the date of the Upper GI Oncology Summit that took place on June 14, 2023, during IGCC2023. The percentage of participants who attended in person is shown in Table 2. During the meeting, a secret ballot was held with independent voting using a Google form, in accordance with the GRADE grid method; the strength of the recommendation was determined based on a consensus rate of ≥ 70% [4]. When a ≥ 70% consensus was not achieved in the second vote, a third vote was planned after consultation.

**Table 2:** Results of voting process

	Total number of vote (in person/web)	Strongly recommend to do	Weakly recommend to do	Weakly recommend not to do	Strongly recommend not to do	Not graded
Surgery CQ 1						
1st voting	45 (0/45)	7 (16%)	32 (71%)	2 (4%)	0 (0%)	4 (9%)
2nd voting	49 (37/12)	3 (6%)	44 (90%)	2 (4%)	0 (0%)	0 (0%)
Surgery CQ 2						
1st voting	45 (0/45)	3 (7%)	38 (85%)	2 (4%)	0 (0%)	2 (4%)
2nd voting	49 (37/12)	1 (2%)	45 (92%)	2 (4%)	0 (0%)	1 (2%)
Surgery CQ 3						
1st voting	45 (0/45)	28 (62%)	13 (30%)	2 (4%)	0 (0%)	2 (4%)
2nd voting	49 (37/12)	20 (41%)	25 (51%)	3 (6%)	1 (2%)	2 (4%)
3rd voting	43 (0/43)	9 (21%)	33 (77%)	0 (0%)	0 (0%)	1 (2%)
Surgery CQ 4						
1st voting	45 (0/45)	3 (7%)	36 (80%)	3 (7%)	1 (2%)	2 (4%)
2nd voting	49 (37/12)	0 (0%)	43 (88%)	3 (6%)	1 (2%)	2 (4%)
Endoscopy CQ 1						
1st voting	45 (0/45)	1 (2%)	32 (72%)	6 (13%)	2 (4%)	4 (9%)
2nd voting	49 (37/12)	1 (2%)	42 (86%)	6 (12%)	0 (0%)	0 (0%)
Endoscopy CQ 2						
1st voting	45 (0/45)	4 (9%)	35 (78%)	2 (4%)	0 (0%)	4 (9%)
2nd voting	49 (37/12)	4(8%)	45(92%)	0 (0%)	0 (0%)	0 (0%)
Endoscopy CQ 3						
1st voting	45 (0/45)	2 (4%)	35 (78%)	2 (11%)	0 (0%)	4 (7%)
2nd voting	49 (37/12)	8 (16%)	39 (80%)	2 (4%)	0 (0%)	0 (0%)
Medical oncology CQ 1						
1st voting	45 (0/45)	10 (22%)	33 (74%)	1 (2%)	0 (0%)	1 (2%)
2nd voting	49 (37/12)	30 (61%)	18 (37%)	0 (0%)	1 (2%)	0 (0%)
3rd voting	43 (0/43)	17 (40%)	24 (56%)	1 (2%)	1 (2%)	0 (0%)
Medical oncology CQ 2						
1st voting	45 (0/45)	13 (29%)	28 (63%)	2 (4%)	0 (0%)	2 (4%)
2nd voting	49 (37/12)	18 (37%)	28 (57%)	3 (6%)	0 (0%)	0 (0%)
3rd voting	43 (0/43)	14 (33%)	28 (65%)	0 (0%)	1 (2%)	0 (0%)
Medical oncology CQ 3						
1st voting	45 (0/45)	40 (90%)	4 (9%)	0 (0%)	0 (0%)	1 (2%)
2nd voting	49 (37/12)	44 (90%)	5 (10%)	0 (0%)	0 (0%)	0 (0%)

*CQ* clinical question

Finally, the third vote was conducted for the responses to 3 CQs with an agreement rate of 70% or lower in the second vote at the conference. Before the third vote, the following rules were announced to the EPMs.

1. If the agreement rate for a recommendation exceeded 70%, that recommendation statement would become the final statement.
2. If the total agreement rate for “strongly recommend to (not) do” and “weakly recommend to (not) do” exceeded 70%, and the total agreement rates for the opposite were lower than 20%, weak recommendation would become the final recommendation statement.
3. If the consensus in both directions (“To do” and “Not to do”) exceeded 20%, “Not graded” would become the final statement.

## Public hearing and external review

During the Upper GI Oncology Summit held on June 14, 2023, the discussion among EPMs was opened to the audience of the summit. Then based on the comments from the participants, modifications were made to the final versions of the statements.

## Improvement of convenience for users

Publication as a publication on the internet, free of charge (websites of the International Gastric Cancer Association, Japanese Gastric Cancer Association, Minds), public lectures, public relations at meetings of scientific societies/study groups.

## Economic independence

The Upper GI Oncology Summit met the entire expenditure for the preparation and publication of these guidelines and did not receive funding from any enterprises.

## Surgery CQ1

Is the dissection of mediastinal and suprapancreatic lymph node stations required for EGJ cancers with 2–4 cm esophageal invasion?

### Recommendation

It is weakly recommended to dissect the lower mediastinal and suprapancreatic lymph node stations during surgery in patients with EGJ cancers showing an esophageal invasion length of 2–4 cm: (result of voting: 90% [44/49]; strength of evidence D).

### Explanatory note

We conducted a literature search and identified 387 articles from the MEDLINE and 51 articles from the Cochrane library. In addition, we extracted 6 papers by hand-searching. Primary screening of these 444 articles yielded 53 papers for secondary screening. We conducted secondary screening of these 53 articles, which yielded 16 articles (13 reports of single-center retrospective cohort studies, 2 reports of prospective cohort studies, and 1 report of a randomized controlled study [RCT]) [5–20]. We reviewed the overall survival, recurrence-free survival, and postoperative complications which were reported as the outcome measures in these articles in patients with EGJ cancer. The definition of EGJ was based on the Siewert Classification in 13 of these studies [5–12, 15, 16, 18–20] and on the Nishi Classification in 3 of these studies [13, 14, 17].

One Korean retrospective observational study reported a direct comparison of treatment results in relation to the presence/absence of mediastinal or abdominal lymph node dissection, and demonstrated that the hazard ratio (HR) for a 5-year disease-free survival was 1.473 (95%CI 0.678–3.199) in the mediastinal lymph node dissection group, not differing significantly from that in the group without mediastinal lymph node dissection [5]. In regard to the outcomes of abdominal lymph node dissection, dissection of the D2 lymph nodes was associated with an HR for 5-year disease-free survival of 3.174 (95% CI 1.302–7.738), differing significantly from that in the groups in whom dissection of other abdominal lymph nodes had also been conducted, although the dissection included suprapancreatic lymph nodes in all cases [5]. Analysis of the postoperative complications revealed that the incidence of respiratory complications was significantly higher in the mediastinal lymph node dissection group. Considering that this type of complication can be serious, the evidence level for this recommendation was set at D (very weak).

With regard to prospective cohort studies, the joint Japan Esophageal Society-Japan Gastric Cancer Association working panel carried out a study of 358 cases of cT2-4 EGJ cancer (Nishi Classification) to evaluate the metastasis rate in each lymph node station [13]. According to their report, the metastasis rate for each lymph node station in tumors with 2.1–4.0 cm esophageal invasion was [station 110]: 15.3%, [station 111]: 4.2% and [station 112]: 2.5%. On the basis of these data, lower mediastinal lymph node dissection has been recommended [13].

One of the RCTs compared two surgical approaches for EGJ tumors: the left thoracoabdominal and the transhiatal approach. In both the intervention (I) group and the patient (P) group, there was strong indirectness to the PICO (P: Patient, I: Intervention & Exposure, C: Comparison, O: Outcome) set in the analysis [12]. For a meta-analysis of the retrospective observational studies, we used the overall 5-year survival rate. Nine of the 15 retrospective observational studies had sufficient data on overall survival, and meta-analysis was performed [5, 9–11, 14, 15, 17, 18, 20]. The meta-analysis revealed a significantly poorer overall survival in the left thoracoabdominal approach group in terms of the overall survival (HR = 0.592 [95%CI 0.386–0.909]), but the definition of the intervention (I) and patients (P) in these studies for each approach were unclear. There were differences in histological type and length of esophageal invasion. These studies contained strong indirectness. Furthermore, with regard to the recurrence-free survival, three papers reporting such analysis were extracted and subjected to meta-analysis [5, 10, 11]. In this analysis, the left thoracoabdominal approach group showed a significantly poorer outcome in terms of the 5-year recurrence-free survival rate (HR = 0.557 [95%CI 0.404–0.768]), but the indirectness was also strong. All studies were retrospective cohort studies and had a high risk of bias. Hence, the evidence level was set at D (very weak).

With regard to safety, 4 papers reporting postoperative complications were extracted and subjected to a meta-analysis. This showed no significant difference between the groups with and without mediastinal lymph node dissection in terms of the incidence of postoperative complications (HR = 0.970 [95%CI 0.369–2.545]) [5, 6, 8, 13]. However, there were only retrospective cohort studies and because the indirectness to the set PICO was strong in both the intervention (I) group and the patient (P) group, it was difficult to draw a conclusion about the safety based on the results of this systematic review. Therefore, the evidence level was set at D (very weak).

On the basis of these results, taking into account the benefit–risk balance and evidence level, the response to the CQ is as follows: there is weak evidence to recommend dissection of the lower mediastinal and suprapancreatic lymph node stations during surgery in patients with EGJ cancers showing an esophageal invasion length of 2–4 cm.

## Surgery CQ2

Is it recommended to dissect the same lymph node region of EGJ squamous cell carcinoma and EGJ adenocarcinoma?

### Recommendation

It is weakly recommended to conduct a similar degree (location and station) of dissection of lymph nodes, regardless of the histological type, during surgery for cT2 or more advanced EGJ cancers: (result of voting: 92% [45/49]; strength of evidence: D).

### Explanatory note

We conducted a literature search to formulate a response to this CQ and extracted 441 papers from the MEDLINE and 24 papers from the Cochrane library. During the literature search, we attempted to extract papers dealing with mediastinal lymph node dissection in patients with EGJ cancer compliant to the Nishi Classification and/or Siewert Type II. After primary and secondary screening, we conducted a qualitative systematic review of 39 reports of case series [9–14, 17, 18, 20–50]. Of the 39 papers, the Siewert Classification was used in 35 and the Nishi Classification in 4, to define the disease. From the 39 reports, we extracted the mediastinal and abdominal para-aortic (station 16a2) lymph node metastasis rate and the dissection efficacy index which is defined as the incidence of metastasis to a region (%), multiplied by the 5-year survival rate (%) of patients with metastasis to that region and divided by 100, for each histological type as the outcome measures, to formulate a response to this CQ. The details of the location for each lymph node are described in Supplementary Table 2.

Analysis of the mediastinal lymph node metastasis rate according to histological type is summarized in Supplementary Table 3 with the dissection efficacy index, which is defined as the incidence of metastasis to a region (%), multiplied by the 5-year survival rate (%) of patients with metastasis to that region and divided by 100 (the higher the number, the more important to dissect the nodal station). Yamashita et al. conducted a large-scale retrospective study of patients with EGJ cancer with a tumor diameter of 4 cm or less via a nationwide questionnaire survey [35]. The lymph node metastasis rates in pT1-T4 EGJ cancer are described in Supplementary Table 4. However, most studies were retrospective case series and as such are prone to selection bias. Kurokawa et al., conducted a prospective study (jointly conducted by the Japan Gastric Cancer Association and the Japan Esophageal Society), including 358 patients with cT2-T4 EGJ cancer (Nishi Classification) and evaluated the metastasis rate in each lymph node group [13]. The metastasis rates are thoroughly described in Supplementary Table 5. There was no evident difference in the mediastinal lymph node metastasis rates depending on the histological type. Based on these results, they recommend that the extent of mediastinal lymph node dissection be determined according to the length of esophageal invasion.

In regard to the No.16a2 lymph node, the metastasis rate was 1.8%–22.2% (adenocarcinoma 4.8%–23.8%, squamous cell carcinoma 3.2%–16.7%) [9, 12–14, 18, 20, 24, 25, 27, 32, 33, 35, 36, 39, 40, 44, 46, 48], and the dissection efficacy index was 0–4.8 (adenocarcinoma 0–4.8, squamous cell carcinoma 0). However, since most of these studies pertained to retrospective case series, it needs to be borne in mind that the studies are prone to selection biases. In a prospective randomized comparative study (JCOG9502) on two different surgical approaches (left thoracoabdominal versus transhiatal) for patients with junctional/gastric cancer with an esophageal invasion of 3 cm or less, the abdominal para-aortic lymph node (No. 16) metastasis rate in patients with Siewert Type II EGJ cancer was 9.3%. Yamashita et al. conducted a large-scale retrospective study of patients of EGJ cancer with a tumor diameter of 4 cm or less (including cT1–4 cases) via a nationwide questionnaire survey, and reported that the No.16a2 lymph node metastasis rate of pT1–T4 E = G EGJ cancer was 0%–0.8% (adenocarcinoma 0.8%, squamous cell carcinoma 0%) [35]. Kurokawa et al. conducted a prospective study (jointly conducted by the Japan Gastric Cancer Association and the Japan Esophageal Society) in 358 patients with cT2–T4 EGJ cancer (Nishi Classification), and reported that the abdominal para-aortic lymph node (No.16a2) metastasis rate was 4.7% (adenocarcinoma 4.8% and squamous cell carcinoma 3.2%) [13].

Based on the available data and evaluation of the evidence level (taking into consideration the benefit–risk balance and data on the lymph node metastasis rates and dissection efficacy index), the desire of the patients, etc., it is concluded: “It is weakly recommended to conduct a similar degree (location and station) of dissection of lymph nodes, regardless of the histological type, during surgery for cT2 or deeper EGJ cancers.” Regarding the extent of mediastinal lymph node dissection, it is recommended to decide the extent depending on the esophageal invasion length.

## Surgery CQ3

Is minimally invasive surgery recommended for EGJ cancer when a transthoracic approach is indicated?

### Recommendation

It is weakly recommended to conduct thoracoscopic (robotic) esophagectomy in patients with resectable EGJ cancer (adenocarcinoma, squamous cell carcinoma) compared to open esophagectomy when a transthoracic approach is indicated: result of voting: 77% [33/43]; strength of evidence: C).

### Explanatory note

We conducted a literature search to formulate a response to this CQ and extracted 804 papers from the MEDLINE and 143 papers from the Cochrane library. In addition, we extracted 3 papers by hand-searching. We conducted primary screening of these 940 papers and extracted 202 papers for secondary screening. And after secondary screening, we extracted 20 articles (11 reports of retrospective cohort studies and 9 reports of overseas multicenter randomized comparative studies). We then evaluated the 5-year overall survival rate, 5-year recurrence-free survival rate, incidence of postoperative complications, and the postoperative quality of life (QOL) as the outcome measures to respond to this CQ.

As there were no randomized comparative studies of the outcomes of minimally invasive esophagectomy in only patients with resectable EGJ cancer, we extracted studies that included patients with EGJ cancer.

Three studies (including TIME Trial) from Europe (primarily in the Netherlands) were available randomized comparative studies that directly compared the outcomes of thoracotomy-based surgery with minimally invasive esophagectomy, and 5 reports have been published based on these studies (comparing the short-term and long-term outcomes) [51–55]. Meta-analysis of the data reported in three of these papers to determine the risk of postoperative complications [51, 53, 55] revealed that the incidence of postoperative complications was significantly lower following minimally invasive esophagectomy (HR: 0.30 [95%CI 0.19–0.48]). The incidence of pneumonia was significantly lower after minimally invasive esophagectomy (HR: 0.36 [95%CI 0.22–0.58]), whereas no significant between-group difference was observed in the incidence of recurrent laryngeal nerve palsy (HR: 1.18 [95%CI 0.44–3.19]) or anastomotic leakage (HR: 1.32 [95%CI 0.68–2.55]). With regard to long-term survival, there was no significant difference in terms of the 3-year recurrence-free survival rate (HR 0.69 [95%CI 0.39–1.24]) or the 3-year overall survival rate (HR: 0.88 [95%CI 0.54–1.44]) between the groups in the TIME trial [53]. Also in the ROBOT trial that compared open with robot-assisted esophagectomy, there was no significant difference in the 5-year overall survival rate (robot group 41% vs. thoracotomy group 40%) or 5-year recurrence-free survival rate (robot group 42% vs thoracotomy group 43%) [52]. In an analysis of the postoperative QOL in the ROBOT trial, short-term QOL (at the time of discharge and 6 weeks after discharge) was significantly better in the robot group [55]. In the TIME trial, the QOL score at 1 year after surgery was higher in the thoracoscopy group [56].

Two randomized studies (including the multicenter MIRO trial from France) compared the outcomes of hybrid minimally invasive esophagectomy (laparoscope-aided transabdominal approach combined with thoracotomy-based esophagectomy) and thoracotomy-based open surgery. Two studies report on the short-term and long-term outcomes [57, 58] and 1 study on the comparison of QOL [59] have been published. Meta-analysis on postoperative complications showed no significant between-group difference in terms of incidence of postoperative complications between the two approaches (HR 0.44 [95%CI 0.15–1.28]). The incidence of pneumonia was lower in the hybrid minimally invasive esophagectomy group (HR 0.58 [95%CI 0.28–0.98]). The incidence of anastomotic failure did not differ significantly between the two surgical groups (HR 1.41 [95%CI 0.58–3.41]). In the MIRO trial, there was no significant difference in the 5-year overall survival rate (HR: 0.67 [95%CI 0.44–1.01]) or 5-year recurrence-free survival rate (HR: 0.76 [95%CI 0.52–1.11]) between the two surgical groups [57]. In the MIOMIE trial also, there was no significant difference in the 5-year overall survival rate or 5-year recurrence-free survival rate between the two groups [58]. In regard to the QOL, the social activity level and extent of pain alleviation at 2 years after surgery were more favorable in the hybrid minimally invasive esophagectomy group [59].

Based on these data, we conclude that minimally invasive esophagectomy may be associated with a reduced incidence of postoperative complications (particularly pneumonia) and improved postoperative short-time QOL as compared to thoracotomy-based surgery, while there appears to be no significant difference in long-term survival between these two types of procedures. As the studies included a sufficient number of patients with EGJ cancer, the evidence level for this recommendation is determined as C, with the indirectness of the evidence taken into consideration.

Eleven reports were observational studies that compared the outcomes of thoracotomy-based surgery with those of minimally invasive esophagectomy in patients with EGJ cancer [60–70]. A meta-analysis of the incidence of postoperative complications including 6 studies [60–66] revealed no significant inter-group difference in terms of the incidence of postoperative complications (HR: 0.98 [95%CI: 0.89–1.07]). There were 6 observational studies that reported long-term survival [63–67]. One of them reported more favorable outcomes of minimally invasive surgery after adjustments for risk factors. Although several studies [67, 68] showed more favorable outcomes in terms of the QOL after minimally invasive surgery, there was no difference in other reports [69, 70]. Because all these reports were retrospective cohort studies with a high risk of selection bias and because randomized studies are available, the results published from these studies seem less valid.

On the basis of these results and considering the benefit–risk balance, patient wishes, evidence level, our response to this CQ is as follows: “It is weakly recommended to conduct thoracoscopic (robotic) esophagectomy in patients with resectable EGJ cancer (adenocarcinoma, squamous cell carcinoma) with compared to open esophagectomy when a transthoracic approach is indicated.”

## Surgery CQ4

Is surgical resection recommended for gastroesophageal junction cancer with oligo-metastasis?

### Recommendation

It is weakly recommended to perform surgical resection after chemotherapy in carefully selected EGJ cancer patients presenting with oligo-metastases: (result of voting: 88% [43/49]; strength of evidence: D).

### Explanatory note

Although there is no uniform definition for oligo-metastases, the Oligo Metastatic Esophagogastric Cancer (OMEC) Project defined oligo-metastases as the presence of only 1 or 2 foci of metastasis affecting one or two organs (liver, lung, para-aortic lymph node, adrenal gland, soft tissue, bone), with no progression (except possible increase in size) apparent after systemic treatment for about 18 weeks [71]. In the other study, oligo-metastases were defined as one to three extracranial metastatic lesions, and a disease-free interval from primary tumor development to metastases of longer than 6 months (with the exception of synchronous colorectal liver metastases) [72].

To formulate a response to this CQ, we defined increase in survival after surgical resection of the oligo-metastases as the outcome measure. We conducted a literature search in the MEDLINE using the keywords, “esophagogastric junction cancer,” “oligometastasis,” “gastric cancer,” “metastasis,” and “surgical resection.” We also conducted a search of the Cochrane library using similar keywords. We extracted 118 articles based on a primary screening and 17 articles by secondary screening of the relevant articles published until August 2022.

This CQ includes two procedures, i.e., (1) simultaneous surgical resection of primary and metastatic lesions in EGJ cancer patients with synchronous oligo-metastases, and (2) resection of metastatic lesions in EGJ cancer patients with metachronous oligo-metastases.

First, we examined whether simultaneous surgical resection of EGJ cancer with synchronous oligo-metastases might prolong survival or not. The AIO-FLOT3 trial was the only prospective interventional study that was designed to evaluate the significance of surgical resection for cases with synchronous oligo-metastases. Of the 252 patients registered, 116 patients (46%) had EGJ cancer [73]. In that trial, the prognosis after chemotherapy was evaluated according to three groups: a group without distant metastasis (Group A), a group with oligo-metastasis affecting only one organ (para-aortic lymph node, liver, lung, etc.) (Group B), and a group with numerous metastases, exceeding the definition of oligo- metastases (Group C). In Group B, surgery was performed if R0 resection was felt to be possible after at least 4 courses of FLOT (5-fluorouracil, leucovorin, oxaliplatin, and docetaxel) therapy. In Group C, FLOT therapy was applied to all patients. The median survival was 22.9 months in Group B and 10.7 months in Group C, indicating a more favorable outcome of surgical resection after chemotherapy in cases of oligo-metastasis. However, patient selection likely plays a major role leading to better survival rates in responders to chemotherapy in this non-randomized study. Serious postoperative complications developed in 3 patients (8.3%) of Group B, while there were no cases of postoperative death during hospitalization.

An increasing number of reports of retrospective observational studies of the effects of surgical resection or other local ablative techniques in cases of oligo-metastases have recently been reported. However, many of these reports pertain to studies in patients with gastric or esophageal cancer, with limited number of patients with EGJ cancer. Therefore, studies in which the proportion of patients with EGJ cases was unclear were excluded from our evaluation. Kroese et al. conducted a single-center retrospective observational study in the Netherlands, in which the outcomes among uncombined local therapy (surgical resection, radiotherapy, or radiofrequency ablation), local therapy plus chemotherapy, and chemotherapy alone were compared in 85 patients with esophageal/gastric cancers (including 14 cases of EGJ cancer) having oligo-metastases [74]. The overall survival was the longest in the local therapy plus chemotherapy group (median 38 months in this group vs. 17 months in the uncombined local therapy group and 18 months in the chemotherapy alone group). Kroese et al. also conducted an international cooperative retrospective observational study in Switzerland and the Netherlands, comparing the outcomes among uncombined local therapy (surgical resection, radiotherapy or radiofrequency ablation), local therapy plus chemotherapy, and chemotherapy alone in 200 patients with esophageal/gastric cancers (including 32 cases of EGJ cancer) having oligo-metastasis [75]. Consistent with the results of the previously mentioned trial above, the overall survival was longest in the local therapy plus chemotherapy group (median 35 months in this group vs. 24 months in the uncombined local therapy group and 13 months in the chemotherapy alone group). At present, the RENAISSANCE (AIO-FLOTS) trial is underway as a multicenter prospective randomized phase III trial in which gastric or EGJ cancer patients with oligo-metastases are assigned after 4 courses of FLOT therapy to the surgical resection group and chemotherapy group at a ratio of 1:1. The results of this trial are awaited [76].

Next, we evaluated articles to determine whether surgical resection of metastatic lesions in patients with metachronous oligo-metastases might prolong survival or not. Until date, no randomized comparative studies or prospective interventional studies have been published on surgical resection of metachronous oligo-metastases in patients with EGJ cancer. In the aforementioned retrospective studies by Kroese et al., metachronous oligo-metastases were present in 50.1% (43/85 cases) [74] or 52.0% (104/200 cases) [75] of the subjects. Although no analysis of the treatment outcomes limited to patients with metachronous oligo-metastases was performed in these studies, the data from these studies seem to endorse the usefulness of combining local treatment (primarily surgery) with chemotherapy.

Apostolidi et al. conducted a single-center retrospective observational study in Germany to evaluate the pattern of recurrence and prognosis in gastric/esophageal cancer patients who had undergone radical resection plus perioperative chemotherapy [77]. In that study, patients with EGJ cancer represented 61.3% (73/119 cases) of the study group. The overall survival was significantly prolonged in patients who had undergone surgery or radiotherapy for recurrent lesions as compared to patients who had received no such treatment (median 35.2 months [95% CI 8.7–18.9] vs. 7.8 months [95% CI 4.9–7.8]). However, since the patients enrolled to this study included some patients with other types of metastases than oligo-metastasis, the reported results need to be interpreted with caution.

There were studies suggesting that surgical treatment of EGJ cancer patients with oligo-metastases can lead to prolongation of the overall survival; however, only one study was a non-randomized prospective trial (the others were retrospective observational studies), and there was no report of studies limited to patients with EGJ cancer. Selection bias and relatively small sample sizes would be problematic here. Moreover, it remains unclear if additive surgery does lead to a better survival compared to radiation or other ablative treatments. Furthermore, the risk of postoperative complications and reduced quality of life post-gastrectomy are important issues to take into consideration.

Taken together, our response to the CQ is as follows: “It is weakly recommended to conduct a surgical resection after chemotherapy in carefully selected EGJ cancer patients presenting with oligo-metastases.”

## Endoscopy CQ1

Is white light endoscopy (WLE) alone recommended for the detection of superficial neoplasia (cancer/ high grade dysplasia (HGD)) at the gastroesophageal junction zone (GEJZ) as compared to WLE combined with image-enhanced endoscopy?

### Recommendation

It is weakly recommended to use WLE alone for the detection of esophagogastric junction adenocarcinoma, except in high-risk patients such as those with Barrett’s esophagus: (result of voting 86% [42/49]; strength of evidence: C).

### Explanatory note

We conducted a search of the literature to answer this CQ. In the primary screening, 419 articles from the MEDLINE and 83 articles from the Cochrane library were selected. Of these, 74 articles were subjected to secondary screening. Finally, we conducted a qualitative systematic review (SR) of 4 articles describing randomized controlled trials (RCTs) [78–81] and 3 articles containing systematic reviews [82–84]. All of these articles describe the results of comparison of WLE and image-enhanced endoscopy [using dyes or acetic acid, narrow-band imaging (NBI), blue laser imaging (BLI), auto-fluorescence imaging** (**AFI), etc.)].

To determine the costs and benefits of the diagnosis by WLE, the following outcomes were considered: (1) the diagnostic accuracy for cancer/HGD; (2) the patient burden associated with the endoscopic examination; (3) the examination cost; and (4) the incidence of adverse events. In regard to (1), the detectability (sensitivity) of HGD/Ca in each testing modality was used as the outcome. For (2) to (4), a discussion is difficult, because the articles did not contain the relevant information.

Of the studies included in the reviews, all the RCTs were conducted in patients with histologically proven Barrett’s esophagus ≥ 2 cm in length, and some even had exclusion criteria that made patients with Barret’s esophagus < 1 cm in length [78–81]. Although it is also difficult to identify the details of the study patients included in systematic reviews, most of the study patients had Barrett’s esophagus. Therefore, the significant indirectness was found between the background of the studies and the population for this CQ, that is, patients undergoing upper gastrointestinal endoscopy [82–84].

The 4 articles on the RCTs compared random biopsy under WLE with target biopsy under AFI-NBI (2 articles), under NBI (1 article), and under acetic acid chromoendoscopy (1 article) [78–81]. In all these studies, the image-enhanced endoscopy failed to demonstrate improvement of the tumor detection, as compared with random biopsy, but showed a higher rate of tumor detection as compared with WLE alone [78–81]. The 3 articles containing systematic review article [82–84] reported a higher tumor detection rate with image-enhanced endoscopy than with WLE. Moreover, one of the systematic review articles, reported by the American Society for Gastrointestinal Endoscopy (ASGE) Technology Committee, reported that only the use of acetic acid and NBI met the Preservation and Incorporation of Valuable Endoscopic Innovations (PIVI) threshold (a per-patient sensitivity of ≥ 90% and negative predictive value of ≥ 98%), which is an acceptable performance threshold to eliminate the need for biopsy [84].

As mentioned above, image-enhanced endoscopy (IEE) using NBI or acetic acid may improve the diagnosis rate of cancer of the esophagogastric junction. However, all the studies selected from the present search were conducted in patients with tumors in Barrett’s esophagus. Therefore, there is no direct evidence as to whether performance of image-enhanced endoscopy in all patients who are eligible for esophagogastroduodenoscopy (EGD) screening might have an add-on effect on the detection rate of esophagogastric junction cancer as compared with WLE alone. While the risk of adverse events associated with the addition of image-enhanced endoscopy to WLE is considered to be low, however, be a slight increase in the patient burden, as a result of the longer examination time and higher medical cost. Therefore, it is considered inappropriate to routinely perform the examination in all patients, including low-risk patients without Barrett’s esophagus. Thus, our recommendation for the CQ1 might be put forth as follows: “It is weakly recommended to use WLE alone for the detection of esophagogastric junction adenocarcinoma, except in high-risk patients such as those with Barrett’s esophagus.”

## Endoscopy CQ2

Is WLE useful to determine the extent of superficial neoplasia　(cancer/HGD) at the GEJZ?

### Recommendation

It is weakly recommended to use other modalities, such as IEE, in addition to WLE alone, for determining the lateral extent of superficial neoplasia at the GEJZ: (result of voting: 92% [45/49]; strength of evidence: C).

### Explanatory note

There are no studies that have verified the diagnostic accuracy of WLE alone for evaluating tumor extension of superficial neoplasia at the esophagogastric junction. Therefore, its precise diagnostic accuracy remains unknown. In addition, there have been no reports of prospective, RCTs comparing WLE with other modalities. Some exploratory studies that investigated several imaging modalities reported that BLI, confocal laser endomicroscopy (CLE), and endoscopy with acetic acid chromoendoscopy may be more effective than WLE for diagnosing the extent of Barrett’s esophagus-associated neoplasm.

One study compared high-definition WLE (HD-WLE) and BLI to visualize the lesions and to delineate lesions using images of early Barrett’s neoplasia obtained with each imaging modality. The study reported that BLI images were significantly better than WLE images for visualizing lesions and allowed better delineation of lesions [85]. In addition, a study that examined the correspondence between pathological diagnosis and endoscopic images obtained by WLE and 1.5% acetic acid chromoendoscopy showed that the diagnostic rate for extension of Barrett’s cancer under the squamous epithelium was 100% by acetic acid chromoendoscopy, which was better than the rate of 50% obtained by WLE [86]. These results suggest that BLI and acetic acid chromoendoscopy are effective for evaluating tumor extension in cases of Barrett’s esophagus-associated neoplasia. However, the study design in both studies had limitations: neither was a direct patient-to-patient comparison of endoscopic diagnostic accuracy, and the evaluation was limited to a retrospective comparison of extracted images. One prospective study that examined the role of CLE for assessing lateral tumor extension and tumor extension under the squamous epithelium in cases of Barrett’s esophagus-associated neoplasia (HGD and esophageal adenocarcinoma) reported that further evaluation by CLE after lesion evaluation by HD-WLE and NBI revealed additional concomitant lesions, lateral tumor extension, or tumor extension under the squamous epithelium in 18% of all patients [87].

Based on the above reports, WLE alone is considered as insufficient for diagnosing lateral extension of superficial neoplasia at the esophagogastric junction, especially Barrett’s esophagus-associated neoplasia, and the diagnostic accuracy of WLE could potentially be improved by combining it with other diagnostic modalities, such as IEE. However, there are no studies that provide sufficient evidence owing to limitations of the study designs. In addition, studies that evaluated BLI, acetic acid chromoendoscopy, and CLE are limited to one article for each of these modalities. Therefore, it has not yet been identified which modality may be best combined with WLE. Thus, our response to CQ 2 is as follows: “It is weakly recommended to use other modalities, such as IEE, in addition to WLE alone, for determining the lateral extent of superficial neoplasia at the GEJZ. (Evidence Level C)”.

In clinical practice, other modalities, especially IEE, have already been combined with WLE to determine the extent of lesions comprehensively. To clarify the best diagnostic method, it would be desirable to conduct patient-to-patient comparison studies to identify the most effective modality and its diagnostic accuracy when combined with WLE.

## Endoscopy CQ3

What are the criterion for curative resection of neoplasia at the GEJZ?

### Recommendation

There is data to recommend endoscopic resection with curative intent for superficial neoplasia of GEJZ. It is weakly recommended that endoscopic resection is considered as curative if the following criteria are fulfilled: the tumor is either (i) an intramucosal carcinoma, or (ii) carcinoma with a submucosal invasion depth of ≤ 500 μm and the tumor diameter ≤ 3 cm; negative resection margins; with no evidence of lymphovascular invasion or poorly differentiated components: (result of voting: 80% [39/49]; strength of evidence: C).

### Explanatory note

For this CQ, the lesion is defined as GEJZ adenocarcinoma or Barrett’s adenocarcinoma (BA). In general, GEJZ adenocarcinoma is considered as type II according to the Siewert Classification [88], i.e., adenocarcinoma located between 1 cm above and 2 cm below the esophagogastric junction, including BA. Especially in Japan, BAs mainly arise from short-segment Barrett’s esophagus [89]. Therefore, it is expected that many GEJZ adenocarcinoma are included in the articles analyzing BA. Thus, we included BA in our literature search.

We conducted a literature search to answer this CQ. In the primary screening, we selected 79 articles from the Cochrane library database and 356 articles from MEDLINE. Then of these 435 articles, we subjected 82 articles to secondary screening. Finally, a qualitative systematic review of 27 articles was conducted, all of which were reports of retrospective observational studies.

In regard to lymph node metastases, 16 articles evaluated the results of treatment in patients with lymph node metastases who underwent surgical resection (including additional surgical resection after endoscopic resection) [90–105]. Based on these reports, the lymph node metastasis rate was 19.3% (101 of 523 patients), but the characteristics of the study patients varied greatly among the articles. None of the articles reported the lymph node metastasis rate by category. However, in one of the articles, a multicenter study calculated the metastasis rate by category by combining patients followed-up for more than 5 years without treatment after endoscopic resection and patients who had undergone surgical resection [93]. In that study, the metastasis rate was 0% in patients with intramucosal carcinoma, negative lymphovascular invasion, and no poorly differentiated components, but 60.0% (9 of 15 patients) in patients with the deep muscularis mucosae invasion with lymphovascular invasion or poorly differentiated components. In addition, the metastasis rate was 0% (0 of 32 patients) in patients having carcinoma with a submucosal invasion depth of ≤ 500 μm, tumor diameter of ≤ 3 cm, negative lymphovascular invasion, and no poorly differentiated components. Based on the above findings, the risk of metastasis is considered to be low in tumors that are (i) intramucosal carcinomas or (ii) carcinomas with a submucosal invasion depth of ≤ 500 μm; tumor diameter of ≤ 3 cm; no evidence of lymphovascular invasion or poorly differentiated components.

Regarding the overall survival rate, the majority of articles calculated the overall survival rate for all patients who had undergone endoscopic resection. The reported 5-year overall survival rate after endoscopic resection was 81%–100% in 9 articles [90–95, 100, 106, 107], excluding studies with a short follow-up period. In one report of a multicenter study, the overall survival rate was calculated by category: the 5-year overall survival rate was 93.9% in low-risk patients (meeting the above criteria) who underwent no additional surgical resection after endoscopic resection, 77.7% in high-risk patients (not meeting the above criteria) who had undergone additional surgical resection after endoscopic resection, and 81.6% in high-risk patients who had not undergone additional surgical resection [89].

None of the articles calculated the recurrence-free survival rate, but five articles calculated the disease-specific survival rate for all patients who had undergone endoscopic resection [91, 92, 94, 95, 107]. The 5-year disease-specific survival rate was 98.4%–100% in these articles [91, 92, 94, 95, 107], and cancer-related deaths corresponded to the abovementioned high risk. In the abovementioned multicenter retrospective study, the disease-specific survival rate was calculated by category. The 5-year disease-specific survival rates after endoscopic resection were 100%, 94.4%, and 92.8%, respectively, for low-risk patients without additional surgical resection, high-risk patients with additional surgical resection, and high-risk patients without additional surgical resection [89].

Adverse events associated with endoscopic resection were evaluated in 21 studies [90, 92, 94, 95, 97–100, 102, 103, 106–115]. The reported adverse events in these articles were combined: the incidence was 1.9% (31 of 1610 patients) for intraoperative perforation, 1.1% (19 of 1610 patients) for intraoperative bleeding, 3.0% (48 of 1610 patients) for postoperative bleeding, 0.06% (1 of 1610 patients) for pneumonia, and 7.1% (114 of 1610 patients) for stenosis. However, serious adverse events requiring emergency surgery developed in only 2 patients (0.1%) who had intraoperative bleeding.

In summary, a good long-term prognosis can be achieved, with no reports of metastases within the above low-risk criteria or cancer-related deaths after endoscopic resection. In addition, although certain adverse events were observed in association with endoscopic resection, the incidence of serious adverse events requiring emergency surgery was extremely low (0.1%). The abovementioned low-risk patients, who did not undergo additional surgical resection and the treatment was completed with endoscopic resection, which is a minimally invasive treatment, have great benefits. Therefore, our response for CQ 3 is as follows: “There is data to recommend endoscopic resection with curative intent for superficial neoplasia of GEJZ. It is weakly recommended that endoscopic resection is considered as curative if the following criteria are fulfilled: the tumor is either (i) an intramucosal carcinoma, or (ii) carcinoma with a submucosal invasion depth of ≤ 500 μm and the tumor diameter ≤ 3 cm; negative resection margins; with no evidence of lymphovascular invasion or poorly differentiated components. (Evidence Level C)” On the other hand, all studies conducted to date are retrospective observational studies, and the sample size in each category is not sufficient in the study to analyze patients by reference category [93]. In particular, the number of patients having carcinoma with a submucosal invasion depth of ≤ 500 μm, tumor diameter of ≤ 3 cm, negative lymphovascular invasion, and no poorly differentiated components is small (32 cases), and some panelists raised a caution regarding this category as curative. Thus, further studies are still demanded in this field.

## Medical oncology CQ1

Is it recommended to adopt chemotherapy for gastric adenocarcinoma to esophageal adenocarcinoma and esophagogastric junction cancer?

### Recommendation

It is weakly recommended to use the same chemotherapy regimens as those established for patients with unresectable advanced or recurrent gastric adenocarcinoma in patients with esophagogastric junction adenocarcinoma and esophageal adenocarcinoma: (result of voting: strongly recommend to do 40% [17/43], weakly recommend to do 56% [24/43]; strength of evidence: C).

### Explanatory note

We conducted a literature search to answer this CQ and extracted 436 articles from the MEDLINE and 378 articles from the Cochrane library. After primary and secondary screening of the articles, we selected 19 research articles [116–134] and reviewed to compare the overall survival, progression-free survival, response rate, and incidence of adverse events. The articles included 1 meta-analysis, 14 reports of randomized controlled trials, 2 reports of single-arm phase II studies, and 2 reports of retrospective studies.

To analyze the best evidence to answer this CQ, we selected studies including subgroup analyses focusing on the esophagogastric junction adenocarcinoma or esophageal adenocarcinoma among studies in patients with esophagogastric junction adenocarcinoma/esophageal adenocarcinoma/gastric adenocarcinoma (hereinafter referred to as upper gastrointestinal tract cancers). Of the articles selected from the screening, we extracted 18 articles [116–122, 124–134] on esophagogastric junction adenocarcinoma and 4 articles [123, 127, 129, 133] on esophageal adenocarcinoma.

The proportion of patients with esophagogastric junction adenocarcinoma and esophageal adenocarcinoma among all patients in the studies ranged from 8% to 43% and 8.8% to 27%, respectively. The reported overall median survival after first-line therapy was 6.9–17.45 months, with a median progression-free survival of 2.0–12.6 months and response rate of 14.8%–74.4%. The reported median survival after second- and subsequent-line therapies was 4–12.5 months, with a median progression-free survival of 2.0–7.0 months and a response rate of 3%–47.9%.

The retrospective studies examined the treatment outcomes of first-line therapies in patients with gastric adenocarcinoma and esophagogastric junction adenocarcinoma. The results showed no significant differences in the overall survival between the two groups [126, 128].

A single-arm phase II study examined the safety and efficacy of first-line combination therapy with capecitabine, oxaliplatin, and docetaxel in HER2 (human epidermal growth factor receptor 2)-negative patients. The proportion of patients with esophagogastric junction adenocarcinoma was 27%, and the overall treatment outcomes in all patients were as follows: response rate, 52.1%; median progression-free survival, 6.9 months; and median overall survival, 12.6 months [125]. In the other study that examined the efficacy and safety of combined therapy with trastuzumab, 5-FU, oxaliplatin, and docetaxel in HER2-positive patients, the proportion of patients with esophagogastric junction adenocarcinoma was 20%, and the overall treatment outcomes in all patients were as follows: response rate, 60%; median progression-free survival, 9.2 months; and median overall survival, 19.4 months [124].

In the phase II randomized controlled DESTINY-Gastric01 trial conducted in HER2-positive patients receiving second- or subsequent-line therapies, the efficacy of trastuzumab deruxtecan (T-DXd) was compared with the attending physician’s choice of systemic chemotherapy [133]. The proportion of patients with esophagogastric junction adenocarcinoma in the T-DXd group was 14%, and the overall treatment outcomes in all patients were as follows: response rate, 43%; median progression-free survival, 5.6 months; and median overall survival, 12.5 months. Subgroup analysis in the T-DXd patients showed a response rate of 50.0% in patients with gastric adenocarcinoma and 60.0% in patients with esophagogastric junction adenocarcinoma. The hazard ratios (HRs) for overall survival in the T-DXd group relative to the chemotherapy group were 0.59 in all patients overall, 0.59 in patients with gastric adenocarcinoma, and 0.68 in patients with esophagogastric junction adenocarcinoma.

Phase III randomized controlled trials on first-line therapy with immune checkpoint inhibitors in HER2-negative patients (KEYNOTE-062, CheckMate 649, ATTRACTION-4 trials, and KEYNOTE-859) were reported recently [2, 122, 131, 132, 134]. The proportion of patients with esophagogastric junction adenocarcinoma in these clinical trials was 30.9–33.1% in the KEYNOTE-062 trial, 17–18% in CheckMate 649 trial, 8–9% in the ATTRACTION-4 trial, and 18.9–23.4% in the KEYNOTE-859 trial. In the CheckMate 649 trial, the proportion of patients with esophageal adenocarcinoma was 12–14%. Subgroup analysis in these clinical trials showed that the HRs (: overall, gastric adenocarcinoma, esophagogastric junction adenocarcinoma, and esophageal adenocarcinoma) for overall survival of combined chemotherapy plus immune checkpoint inhibitor therapy relative to chemotherapy alone were 0.85, 0.81, 0.96, and no data, respectively, in the KEYNOTE-062 trial (combined positive score [CPS] ≥ 1); 0.69, 0.64, 0.82, and 0.73, respectively, in the CheckMate 649 trial (CPS ≥ 5), 0.90, 0.87, 1.00, and no data, respectively, in the ATTRACTION-4 trial; and 0.78, 0.77, 0.74, and data not shown in the KEYNOTE-859 trial; subgroup analysis also showed HRs for progression-free survival of 0.70, 0.71, 0.59, and no data, respectively, in the ATRACTION-4 trial and 0.76, 0.75, 0.78, and no data, in the KEYNOTE-859 trial. For adverse events, there were no results of subgroup analyses for comparison by location of the primary tumor.

In the ToGA trial, where the effect of add-on trastuzumab plus chemotherapy as first-line therapy was examined in HER2-positive patients, the proportion of patients with gastric adenocarcinoma and esophagogastric junction adenocarcinoma was 80%–83% and 17%–20%, respectively [121]. Subgroup analysis showed that the HRs for overall survival of combined chemotherapy plus trastuzumab therapy relative to chemotherapy alone were 0.74 in all patients overall, 0.76 in patients with gastric adenocarcinoma, and 0.67 in patients with esophagogastric junction adenocarcinoma. In the KEYNOTE-811 trial, in which the effect of add-on pembrolizumab to combined chemotherapy plus trastuzumab therapy was verified in HER2-positive patients, the proportion of patients with gastric adenocarcinoma and esophagogastric junction adenocarcinoma was 65.4%–72.2% and 27.8%–34.6%, respectively [119]. According to subgroup analysis, the difference in the response rate between combined chemotherapy plus trastuzumab therapy and combined chemotherapy + trastuzumab + pembrolizumab therapy was 22.7% in all patients overall, 19.9% in patients with gastric adenocarcinoma, and 27.4% in patients with esophagogastric junction adenocarcinoma. Thus, the benefit in response rate to combined chemotherapy + trastuzumab + pembrolizumab therapy was better in patients with EGJ tumors.

In the RAINBOW trial, which was a phase III randomized controlled trial conducted to evaluate second-line therapy, the proportion of patients with gastric adenocarcinoma and esophagogastric junction adenocarcinoma was 79%–80% and 20%–21%, respectively [117]. Subgroup analysis showed that the HRs for overall survival of paclitaxel therapy relative to combined paclitaxel plus ramucirumab therapy was 0.807 in all patients overall, 0.899 in patients with gastric adenocarcinoma, and 0.521 in patients with esophagogastric junction adenocarcinoma. Subgroup analysis also showed corresponding HRs for progression-free survival of 0.635, 0.694, and 0.387, respectively.

In phase III randomized controlled trials conducted to examine the usefulness of systemic chemotherapy relative to placebo or best supportive care (BSC) as second- or subsequent-line therapies, the proportion of patients with gastric adenocarcinoma, esophagogastric junction adenocarcinoma, and esophageal adenocarcinoma was 74%–75%, 25%–26%, and no data, respectively, in the REGARD trial [116]; 71%, 28%–29%, and no data, respectively, in the TAGS trial [130]; and 44%–46%, 32%–38%, and 18%–22% in the COUGAR-02 trial [129]. Subgroup analysis showed HRs for overall survival of chemotherapy relative to placebo or BSC of 0.776 in all patients overall, 0.823 in patients with gastric adenocarcinoma, 0.756 in patients with esophagogastric junction adenocarcinoma, unknown (no data) in patients with esophageal adenocarcinoma patients in the REGARD trial, 0.69, 0.67, 0.75, and unknown (no data), respectively, in the TAGS trial, and 0.67, 0.73, 0.56, and 0.73, respectively, in the COUGAR-02 trial. Subgroup analysis also showed corresponding HRs for progression-free survival of 0.483, 0.513, 0.386, and unknown (no data), respectively, in the REGARD trial.

In regard to integrated analysis, there was an integrated study of 4 randomized controlled trials using individual patient data. Of all 2110 patients, the proportion of patients with gastric adenocarcinoma, esophagogastric junction adenocarcinoma, and esophageal adenocarcinoma was 47%, 26%, and 27%, respectively [127]. The median overall survival was 9.1 months in all patients overall, 8.7 months in patients with gastric adenocarcinoma, 9.3 months in patients with esophagogastric junction adenocarcinoma, and 9.5 months in patients with esophageal adenocarcinoma. The response rate was 35.6% in patients with gastric adenocarcinoma, 41.1% in patients with esophagogastric junction adenocarcinoma, and 44.1% in patients with esophageal adenocarcinoma. For adverse events, there were no differences in the incidence of adverse events by the location of the primary tumor.

Based on the above findings, which were the results of subgroup analyses, the outcome in patients with esophagogastric junction adenocarcinoma or esophageal adenocarcinoma was not different from that in patients with gastric adenocarcinoma. Therefore, the response to CQ1 is as follows: “It is weakly recommended to use the same chemotherapy regimens as those established for patients with unresectable advanced or recurrent gastric adenocarcinoma in patients with esophagogastric junction adenocarcinoma and esophageal adenocarcinoma.”

## Medical oncology CQ2

What is the optimal perioperative treatment for resectable, locally advanced esophagogastric junction cancer?

### Recommendation

Based on the both Eastern and Western evidence, it is weakly recommended to provide perioperative chemotherapy or neoadjuvant chemoradiotherapy for patients with resectable advanced esophagogastric junction cancer. However, the upfront surgery followed by adjuvant chemotherapy may also be acceptable for patients with advanced esophagogastric junction cancer, as for patients with advanced gastric cancer: (rate of consensus: (result of voting: strongly recommend to do 33% [14/43], weakly recommend to do 65% [28/43]; strength of evidence: C).

### Explanatory note

We conducted a literature search to formulate a response to this CQ and extracted a total of 1237 articles, consisting of 668 articles from the MEDLINE and 871 articles from the Cochrane library. A total of 51 articles were selected after the primary screening, and 17 articles through secondary screening. There were very few studies targeting only patients with resectable esophagogastric junction cancer, and in most of the studies selected, the study population included patients with esophageal cancer or gastric cancer. Therefore, we extracted studies that included patients with esophagogastric junction cancer in the study population. And then we narrowed down the articles to studies that mentioned esophagogastric junction cancer in subgroup analyses, etc., and classified the studies by the perioperative therapeutic intervention used, into 1 report of neoadjuvant chemotherapy (randomized phase III trial [135]), 5 reports of perioperative chemotherapy (including 4 of randomized phase III trials [136–139], and 1 of a randomized phase II/III trial [140]), 6 reports of perioperative chemo-(immune)-radiotherapy (including 1 of a randomized phase III trial [141], 2 of randomized phase II trials [142, 143], 1 of a single-arm phase II trial [144], 1 of a single-arm phase Ib/II trial [145], and 1 of a retrospective study [146]), 2 reports of adjuvant chemotherapy (including 2 of randomized phase III studies [139, 147]), 3 reports of adjuvant chemoradiotherapy (including 2 of randomized phase III studies [148, 149] and 1 of a retrospective study [150]), and 1 report of adjuvant immunotherapy (randomized phase III controlled trial [151]). Studies focusing only on patients with esophagogastric junction cancer (including Siewert type III cancer) were 3 articles [141, 143, 145]. In other studies, the proportion of patients with esophagogastric junction cancer ranged from 2 to 64%.

We reviewed the articles to compare the overall survival (OS), progression-free survival (PFS), response rate, incidence of adverse events, and proportion of patients with surgical complications specified as the study outcomes. However, there were no studies that included patients with esophageal cancer and gastric cancer which directly mentioned the proportion of patients with adverse events or surgical complications among the patients with esophagogastric junction cancer.

The POET trial was a randomized phase III study conducted in patients with esophagogastric junction cancer (including Siewert Type I and III), with the 3-year OS set as the primary endpoint, to evaluate adding of neoadjuvant chemoradiotherapy (CRT) (combined cisplatin plus etoposide therapy with irradiation at 30 Gy) to induction chemotherapy (5-FU, leucovorin and cisplatin [PLF]) [141]. The trial was prematurely terminated due to poor accrual, with a 3-year OS of 47.4% in the CRT group, better than that (27.7%) in the neoadjuvant chemotherapy group, although the difference was not statistically significant (HR of 0.67, 95%CI of 0.41–1.07, *P* = 0.07). On the other hand, the postoperative mortality was 10.2% in the CRT group, higher than that (3.8%) in the neoadjuvant chemotherapy alone group. After long-term follow-up, the neoadjuvant CRT group tended to show better results, in both patients with Siewert Type I (HR of 0.71, 95%CI of 0.39–1.29) and Type II (HR of 0.60, 95%CI of 0.32–1.14) cancer. Also, a randomized phase II study conducted in Asia to examine the effect of add-on neoadjuvant CRT (capecitabine plus oxaliplatin (CAPOX) therapy plus radiation therapy (RT) at 45 Gy) to surgery plus adjuvant CAPOX therapy [9]^)^ showed a trend toward improved disease-free survival (DFS), which was set as the primary endpoint in the neoadjuvant CRT group. Regarding the OS, which was set as the secondary endpoint, a significantly better OS was observed in the neoadjuvant CRT arm. Based on these results, it can be said that a trend toward superiority of neoadjuvant CRT over upfront surgery was observed, as in the CROSS trial. However, it was still difficult to determine the superiority of neoadjuvant CRT over neoadjuvant chemotherapy. The MC1541 trial was a single-arm phase Ib/II study conducted to comparatively evaluate the effect of neoadjuvant CRT using the CROSS regimen with chemo-immuno-radiotherapy using the CROSS regimen combined with pembrolizumab [145]. The primary endpoint (pathologic complete response [pCR] rate) was not achieved, and no significant difference in the PFS (*P* = 0.409) was observed as compared with that in the propensity-score-matched group receiving neoadjuvant CRT (CROSS regimen). It has been suggested that the effect could be enhanced in a population with a tumor PD-L1 CPS of ≥ 10. Therefore, further studies including using biomarker evaluation are needed.

Based on the results of the FLOT-4 trial, FLOT therapy has become the standard perioperative chemotherapy regimen for gastric cancer in Western countries. A subgroup analysis to compare the OS showed no interactions between patients with esophagogastric junction cancer and gastric cancer. Therefore, the efficacy of perioperative FLOT therapy can be also expected in patients with esophagogastric junction cancer, as in patients with gastric cancer [138]. In the RESOLVE trial conducted in East Asia, perioperative SOX (S-1 plus oxaliplatin) therapy was found to be superior to resection plus adjuvant CAPOX therapy in terms of the 3-year DFS. In addition, a subgroup analysis showed no impact of the tumor location on the results obtained [139]. In both studies, patients with gastric cancer accounted for more than a half of the study subjects. However, perioperative chemotherapy can also be expected to be effective in patients with esophagogastric junction cancer. A large-scale study was conducted in patients with gastric or esophageal cancer in the early 2000s to examine the significance of neoadjuvant chemotherapy, with upfront surgery used as the standard therapy. The study did not specify the results in patients with esophagogastric junction cancer, and therefore cannot be used as a reference in formulating the response to this CQ. In the PRODIGY trial [135], conducted mostly in South Korea, the proportion of patients with esophagogastric junction cancer was small (7%, *n = *16). Thus, the efficacy of neoadjuvant chemotherapy in patients with esophagogastric junction cancer needs to be further examined.

In East Asia, upfront surgery followed by adjuvant chemotherapy is the standard treatment for gastric cancer. In the CLASSIC trial [147], which was conducted to examine the efficacy of adjuvant CAPOX therapy, the proportion of patients with esophagogastric junction cancer was only 2% (*n = *24). Thus, there is a lack of direct evidence of the efficacy. However, based on the lack of evidence in East Asia and considering that evidence for gastric cancer is often extrapolated to patients with esophagogastric junction cancer, upfront surgery followed by adjuvant chemotherapy may also be recommended for patients with esophagogastric junction cancer.

In the RESOLVE trial, the proportion of patients with esophagogastric junction cancer was 36% (*n = *123). The trial showed non-inferiority of adjuvant SOX therapy to adjuvant CAPOX therapy [139], but the comparison was made between two adjuvant chemotherapy regimens. The effect of adjuvant chemoradiotherapy was examined in the CRITICS [148] and CALGB80101 [149] trials. But, in these trials also, the proportion of patients with esophagogastric junction cancer was limited to 15–25%, and furthermore, the limited number of patients in whom the adjuvant CRT could be completed also precluded adequate evaluation of the efficacy. In regard to adjuvant immunotherapy, the KEYNOTE-577 trial reported that adjuvant nivolumab therapy was effective for prolonging the DFS in non-pCR patients who had received neoadjuvant CRT as compared with those who had received placebo [151]. In the KEYNOTE-577 trial, the proportion of patients with esophagogastric junction cancer was 40% (*n = *224 for nivolumab and *n = *107 for placebo). However, the point estimate of the HR in the subgroup analysis indicated a lower effectiveness (0.87) in patients with esophagogastric junction cancer as compared with that in patients with lower esophageal cancer (0.61). Furthermore, the effectiveness tended to be lower in patients with adenocarcinoma than in those with squamous cell cancer. Therefore, it appears likely that adjuvant nivolumab therapy exerts limited efficacy in patients with esophagogastric junction cancer. Since no data on the OS were reported from the KEYNOTE-577 trial, evidence to recommend the therapy remains insufficient.

Based on these findings, our response to the CQ is as follows: “It is weakly recommended to provide perioperative chemotherapy or neoadjuvant chemoradiotherapy for patients with resectable advanced esophagogastric junction cancer. However, the upfront surgery followed by adjuvant chemotherapy may also be acceptable for patients with advanced esophagogastric junction cancer, as for patients with advanced gastric cancer.” Standard recommendations differ between the different geographic regions. Based on randomized controlled studies conducted in the Western hemisphere, national and international guidelines from Non-Asian regions give stronger recommendations for perioperative treatment [152].

## Medical oncology CQ3

What biomarkers are recommended to be tested before first line for unresectable case?

### Recommendation

It is strongly recommended to evaluate the expression statuses of HER2, PD-L1 combined positive score (CPS), microsatellite instability (MSI), and claudin 18.2 prior to first-line chemotherapy in patients with unresectable esophagogastric junction adenocarcinoma: (result of voting: 90% [44/49]; strength of evidence: C).

### Explanatory note

We conducted a literature search to formulate a response to this CQ and extracted 435 articles from the MEDLINE and 696 articles from the Cochrane library. After primary and secondary screening, we selected 16 research articles [3, 118, 119, 121, 122, 131–134, 153–159] and evaluated them for evidence on biomarker evaluations and the efficacy of chemotherapies. The articles consisted of 14 reports of randomized controlled trials (including one report of the results of comprehensive analysis of 2 randomized controlled trials and 1 single-arm phase II trial) and 2 reports related to analysis for biomarkers using tumor specimens.

HER2 (human epidermal growth factor receptor 2)-positivity was defined as an IHC (immunohistochemistry) score of 3 + or a positive result of FISH in the ToGA trial [121]. A subgroup analysis of patients with high HER2 expression (either IHC3 + or IHC2 + plus FISH-positive) showed prolonged survival in this subset of patients. Therefore, in clinical practice, administration of chemotherapy including trastuzumab is recommended for patients with IHC3 + or IHC2 + plus FISH-positive tumors. The frequency of HER2-positive tumors (IHC3 + or IHC2 + plus FISH-positive) is reported to be about 21% among patients with advanced or recurrent gastric adenocarcinoma, and 32% among patients with advanced or recurrent esophagogastric junction cancer; thus, there is a trend toward a higher frequency in patients with esophagogastric junction cancer [153]. In the ToGA trial, the proportion of patients with gastric adenocarcinoma and esophagogastric junction adenocarcinoma was about 80% and 20%, respectively. In a subgroup analysis, the HRs for overall survival of chemotherapy combined with trastuzumab therapy versus chemotherapy alone were 0.74 in all patients overall, 0.76 in patients with gastric adenocarcinoma, and 0.67 in patients with esophagogastric junction adenocarcinoma.

PD-L1 CPS is defined as the number of cells expressing PD-L1 in the tumor tissue (tumor cells and immune cells [macrophages and lymphocytes]) divided by the total number of viable tumor cells, multiplied by 100 [118, 119, 131, 134, 154–157]. In the CheckMate649 trial, the superiority of combined chemotherapy plus nivolumab, an anti-PD-1 antibody, over chemotherapy alone as first-line treatment was examined in HER2-negative advanced or recurrent gastric adenocarcinoma/esophagogastric junction adenocarcinoma/esophageal adenocarcinoma [131, 134]. The primary endpoint was progression-free survival and overall survival in patients with a tumor PD-L1 CPS of ≥ 5. In this trial, the expression of PD-L1 was evaluated by the PD-L1 IHC 28–8 pharmDx assay. There were 955 patients with CPS ≥ 5, accounting for about 60% of all the patients enrolled. Patients with CPS ≥ 5 showed significantly prolonged progression-free survival and overall survival and a higher response rate in the combined chemotherapy plus nivolumab group than in the chemotherapy alone group. In this trial, the proportion of patients with gastric adenocarcinoma, esophagogastric junction adenocarcinoma, and esophageal adenocarcinoma was about 70%, 18%, and 12%, respectively. A subgroup analysis in patients with CPS ≥ 5 showed that the HRs for overall survival of combined chemotherapy plus nivolumab relative to chemotherapy alone were 0.69 in all patients overall, 0.64 in patients with gastric adenocarcinoma, 0.82 in patients with esophagogastric junction adenocarcinoma, and 0.73 in patients with esophageal adenocarcinoma.

Pembrolizumab, an anti-PD-1 antibody, has been reported as being highly effective against solid tumors with high microsatellite instability (MSI-high), including esophagogastric junction adenocarcinoma. The reported frequency of MSI-high in advanced gastric adenocarcinoma is 3–5% [160]. The Cancer Genome Atlas (TCGA) reported that the frequency of MSI-high is lower in patients with esophagogastric junction adenocarcinoma than in patients with gastric adenocarcinoma [158]. In addition, a subgroup analysis in the CheckMate649 trial showed that the HRs for overall survival in the combined chemotherapy plus nivolumab group as compared with that in the chemotherapy alone group were 0.38 for MSI-high and 0.78 for microsatellite-stable cases.

The superiority of combined chemotherapy plus zolbetuximab (an anti-claudin 18.2 monoclonal antibody) over chemotherapy plus placebo as first-line treatment was evaluated in patients with claudin 18.2-positive and HER2-negative patients with advanced or recurrent gastric or esophagogastric junction adenocarcinoma (SPOTLIGHT trial and GLOW trial; claudin 18.2 positivity was defined as ≥ 75% tumor cells showing strong-to-moderate staining intensity on IHC) [131, 161]. Significantly prolonged progression-free survival and overall survival, set as the primary endpoints, were observed in the combined chemotherapy plus zolbetuximab group compared with the chemotherapy plus placebo group. Similarly, the GLOW trial met the primary endpoint of progression-free survival and key secondary endpoint of overall survival. In the SPOTLIGHT/ GLOW trial, the proportion of patients with gastric adenocarcinoma and esophagogastric junction adenocarcinoma was about 76%/ 84% and 24%/ 16%, respectively. A subgroup analysis in the SPOTLIGHT/ GLOW trial showed that the HRs for overall survival of combined chemotherapy plus zolbetuximab as compared with chemotherapy plus placebo were 0.75/ 0.77 in all patients, 0.67/ 0.72 in patients with gastric adenocarcinoma, and 1.07/ 1.01 in patients with esophagogastric junction adenocarcinoma [131, 161].

These findings show that HER2, PD-L1 CPS, MSI, and claudin 18.2 are important biomarkers for the selection and efficacy prediction of first-line chemotherapy in patients with unresectable esophagogastric junction adenocarcinoma a, although claudin 18.2 IHC is not currently commercially available. Therefore, our response to CQ is as follows: “There is strong evidence to recommend evaluation of the expression statuses of HER2, PD-L1 CPS, MSI, and claudin 18.2 prior to first-line chemotherapy in patients with unresectable esophagogastric junction adenocarcinoma.”

### Supplementary Information

Below is the link to the electronic supplementary material.

Supplementary file1 (DOCX 38 KB)
