# Supplementary material for: Clinical practice guidelines for esophagogastric junction cancer: Upper GI Oncology Summit 2023
Source: Gastric Cancer. 2024 Feb 22;27(3):401–25. doi: 10.1007/s10120-023-01457-3 (PMC11016517; doi:10.1007/s10120-023-01457-3)
Supplement: Supplementary file 1 — Supplementary file1 (DOCX 38 KB) [file 10120_2023_1457_MOESM1_ESM.docx]

**Supplementari table 1.** Details of literature review

| CQ |  | Key words | Period |
| --- | --- | --- | --- |
| Surgery CQ1 | Is the dissection of mediastinal and suprapancreatic lymph node stations required for EGJ cancers with 2-4 cm esophageal invasion? | Esophagogastric junction cancer, mediastinal, lymph node dissection | January 2005 - July 2022 |
| Surgery CQ2 | Is it recommended to dissect the same lymph node region of EGJ squamous cell carcinoma and EGJ adenocarcinoma? | Esophagogastric junction cancer, lymph node metastasis, histology, difference | January 2005 - July 2022 |
| Surgery CQ3 | Is minimally invasive surgery recommended for EGJ cancer when a transthoracic approach is indicated? | Esophagogastric junction cancer, minimally invasive surgery, esophagogastric junction cancer, robot assisted minimally invasive surgery | January 2005 - July 2022 |
| Surgery CQ4 | Is surgical resection recommended for gastroesophageal junction cancer with oligo metastasis? | Esophagogastric junction cancer, oligometastasis, Gastric cancer, oligometastasis, esophagogastric junction cancer, metastasis, surgical resection | January 2005 - July 2022 |
| Endoscopy CQ1 | Is WLE alone recommended for the detection of superficial neoplasia (cancer/HGD) at the GEJZ as compared to WLE combined with image-enhanced endoscopy? | Esophagogastric (junction), gastroesophageal (junction), gastric cardia, Barrett’s epithelium, adenocarcinoma, neoplasia, dysplasia, detection, endoscopy, narrow band imaging (NBI), blue light Imaging (BLI), Linked Color Imaging (LCI), Image enhanced endoscopy (IEE), acetic acid, magnifying endoscopy | January 2005 - June 2022 |
| Endoscopy CQ2 | Is WLE useful to determine the extent of superficial neoplasia　(cancer/HGD) at the GEJZ? | Esophagogastric (junction), gastroesophageal (junction), gastric cardia, Barrett’s epithelium, adenocarcinoma, neoplasia, dysplasia, diagnosis, endoscopy, demarcation, narrow band imaging (NBI), blue light Imaging (BLI), Linked Color Imaging (LCI), Image enhanced endoscopy (IEE), acetic acid, magnifying endoscopy | January 2005 - June 2022 |
| Endoscopy CQ3 | What are the criterion for curative resection of neoplasia at the GEJZ? | Esophagogastric (junction), gastroesophageal (junction), gastric cardia, Barrett’s epithelium, adenocarcinoma, neoplasia, dysplasia, endoscopic resection, endoscopic mucosal resection (EMR), endoscopic submucosal dissection (ESD), indication, lymph node metastasis, recurrence | January 2005 - June 2022 |
| Medical Oncology CQ1 | Is it recommended to adopt chemotherapy for gastric adenocarcinoma to esophageal adenocarcinoma and esophagogastric junction cancer? | Esophagogastric junction cancer, gastric cancer, esophageal adenocarcinoma, chemotherapy, first-line chemotherapy | January 2005 - August 2022 |
| Medical Oncology CQ2 | What is the optimal perioperative treatment for resectable, locally advanced esophagogastric junction cancer? | Esophagogastric junction cancer, gastric cancer, esophageal adenocarcinoma, chemotherapy, perioperative chemotherapy, adjuvant chemotherapy, neoadjuvant chemotherapy | January 2005 - August 2022 |
| Medical Oncology CQ3 | What biomarkers are recommended to be tested before first-line for unresectable case? | Esophagogastric junction cancer, gastric cancer, esophageal adenocarcinoma, chemotherapy, biomarker, HER2, PD-L1, FGFR2b, CLDN18.2 | January 2005 - August 2022 |
| EGJ, esophagogastric junction; WLE, white light endoscopy; HGD, high grade dysplasia ; GEJZ, gastroesophageal junction zone. | | | |

**Supplementari table 2.** Lymph node stations in esophageal cancer

| Japan esophageal society (12th) | | AJCC (8th) |
| --- | --- | --- |
| Cervical lymph nodes | |  |
| 101 (L/R) | Cervical paraesophageal lymph nodes | 1 R/L (IV†) |
| 102up | Upper deep cervical lymph nodes | IIB† |
| 102 mid | Middle deep cervical lymph nodes | III† |
| 103 | Peripharyngeal lymph nodes | IIA & III† |
| 104 (L/R) | Supraclavicular lymph nodes | IV & VB† |
| Thoracic lymph nodes | |  |
| 105 | Upper thoracic paraesophageal lymph nodes | 8up |
| 106recL | Left recurrent nerve lymph nodes | 2 L |
| 106recR | Right recurrent nerve lymph nodes | 2 R |
| 106pre | Pretracheal lymph nodes | 4 R |
| 106tbL | Left tracheobronchial lymph nodes | 4 L |
| 106tbR | Right tracheobronchial lymph nodes | 4 R |
| 107 | Subcarinal lymph nodes | 7 |
| 108 | Middle thoracic paraesophageal lymph nodes | 8 m |
| 109L | Left main bronchus lymph nodes | 10‡ |
| 109R | Right main bronchus lymph nodes | 10‡ |
| 110 | Lower thoracic paraesophageal lymph nodes | 8lo |
| 111 | Supradiaphragmatic lymph nodes | 15 |
| 112aoA | Anterior thoracic paraaortic lymph nodes | 8 m & 8lo |
| 112aoP | Posterior thoracic paraaortic lymph nodes | 8 m & 8lo |
| 112pul (L/R) | Pulmonary ligament lymph nodes | 9 R/L |
| 113 | Ligamentum arteriosum lymph nodes (Botallo lymph nodes) | 5 |
| Abdominal lymph nodes | |  |
| 1 | Right paracardial lymph nodes | 16 |
| 2 | Left paracardial lymph nodes | 16 |
| 3a | Lesser curvature lymph nodes along the branches of the left gastric artery | 17 |
| 7 | Lymph nodes along the left gastric artery | 17 |
| 8a | Lymph nodes along the common hepatic artery (anterosuperior group) | 18 |
| 9 | Lymph nodes along the celiac artery | 20 |
| 11p | Lymph nodes along the proximal splenic artery | 19 |
| 11d | Lymph nodes along the distal splenic artery | 19 |
| 19 | Infradiaphragmatic lymph nodes | 16 |
| 20 | Lymph nodes in the esophageal hiatus of the diaphragm | 16 |

† AJCC head and neck cancer staging (eighth edition).

‡ AJCC lung cancer staging (eighth edition).

**Supplementary table 3**. Metastatic rate and dissection efficacy index for each lymph node station based on the literature review

| Station | All | Adenocarcinoma | Squamous cell carinoma |
| --- | --- | --- | --- |
| Metastatic rate | | | |
| Superior mediastinal lymph nodes | NA | 0%-15.8% | 0%-8.3% |
| Middle mediastinal lymph nodes | NA | 2%-20% | 4%-31.3% |
| 110 | NA | 3%-12% | 13%-30% |
| 111 | NA | 0% | 4%-8% |
| 112 | NA | 0%-13% | 8%-9% |
| Dissection efficacy index | | | |
| 105 | 0 | 0 | 0 |
| 106 | 0-5.0 | 0 | 2.2-5.0 |
| 107 | 0-2.6 | 2.6 | 1.2 |
| 108 | 0-2.5 | 2.0-2.5 | 2.0-2.4 |
| 109 | 0-3.8 | 0 | 3.8 |
| 110 | 1.1-7.8 | 1.1-2.9 | 3.3-7.8 |
| 111 | 0-1.2 | 0-1.2 | 0-1.1 |
| 112 | 0-1.1 | 0-1.1 | 0-1.1 |
| Location for each station is listed in Supplementary table 2. | | | |

**Supplementary table 4**. Metastatic rate and dissection efficacy index for each lymph node station of esophagogastric junction cancer in a large-scale retrospective study of patients with EGJ cancer with a tumor diameter of 4 cm or less

| Station | Total | Adenocarcinoma | Squamous cell carinoma |
| --- | --- | --- | --- |
| Metastatic rate | | | |
| 105 | 0%-1.1% | 0.3%-0.4% | 0%-1.1% |
| 106 | 0%-5.1% | 0%-0.3% | 0.7%-5.1% |
| 107 | 0%-1.7% | 0%-0.4% | 0%-1.7% |
| 108 | 0.8%-4.0% | 0.8%-1.3% | 1.3%-4.0% |
| 109 | 0%-2.8% | 0%-1.7% | 0%-2.8% |
| 110 | 0.5%-11.9% | 0.5%-5.1% | 2.7%-11.9% |
| 111 | 0.3%-3.4% | 0.3%-1.7% | 0.7%-3.4% |
| 112 | 0%-2.3% | 0.5%-1.3% | 0%-2.3% |
| Dissection efficacy index | | | |
| 107 | 1.2-2.6 | 2.6 | 1.2 |
| 108 | 0-2.5 | 0 | 2.5 |
| 109 | 0-3.8 | 0 | 3.8 |
| 110 | 0-7.8 | 1.9 | 7.8 |
| 111 | 0-1.2 | 1.2 | 0 |
| 112 | 0-1.1 | 0 | 1.1 |
| Location for each station is listed in Supplementary table 2. | | | |

**Supplementary table 5.** Metastatic rate for each lymph node station of esophagogastric junction cancer in a prospective study (jointly conducted by the Japan Gastric Cancer Association and the Japan Esophageal Society)

| Station | Total | Adenocarcinoma | Squamous cell carinoma |
| --- | --- | --- | --- |
| All patients | | | |
| 105 | 1.0% | 1.5% | 0% |
| 106recL | 1.0% | 1.5% | 0% |
| 106recR | 5.1% | 6.0% | 3.2% |
| 107 | 3.1% | 3.0% | 3.2% |
| 108 | 5.1% | 3.0% | 9.7% |
| 109L | 3.1% | 3.0% | 3.2% |
| 109R | 2.0% | 1.5% | 3.2% |
| 110 | 9.3% | 9.0% | 12.9% |
| 111 | 3.4% | 3.7% | 0% |
| 112 | 2.0% | 1.9% | 3.2% |
| Patients with invasion length of 1.1-2.0 cm | | | |
| 110 | 6.4% | NA | NA |
| 111 | 2.2 | NA | NA |
| 112 | 2.2 | NA | NA |
| Patients with invasion length of 2.1-4.0 cm | | | |
| 110 | 15.3% | NA | NA |
| 111 | 4.2% | NA | NA |
| 112 | 2.2% | NA | NA |
| Patients with invasion length of more than 4.0 cm | | | |
| 105 | 3.6% | NA | NA |
| 106recL | 3.6% | NA | NA |
| 106recR | 10.7% | NA | NA |
| 107 | 7.1% | NA | NA |
| 108 | 7.1% | NA | NA |
| 109L | 7.1% | NA | NA |
| 109R | 3.6% | NA | NA |
| 110 | 28.6% | NA | NA |
| 111 | 10.7% | NA | NA |
| 112 | 7.1% | NA | NA |
| Location for each station is listed in Supplementary table 2. | | | |
